# Perception and readiness of community pharmacists in delivering palliative care services in Saudi Arabia: a new role in the game

**DOI:** 10.3389/fphar.2025.1646531

**Published:** 2025-11-25

**Authors:** Sultan M. Alshahrani

**Affiliations:** Department of Clinical Pharmacy, College of Pharmacy, King Khalid University, Abha, Saudi Arabia

**Keywords:** community pharmacists, palliative care, Saudi Arabia, readiness, knowledge, attitudes

## Abstract

**Background:**

Palliative care (PC) improves the quality of life for patients with life-limiting illnesses; however, the involvement of community pharmacists in Saudi Arabia remains underexplored. This study evaluates community pharmacists’ perspectives, readiness, and barriers to providing pharmaceutical care services within the Vision 2030 healthcare transformation framework.

**Methods:**

A cross-sectional survey was conducted among 267 licensed community pharmacists in the Aseer region of Saudi Arabia using convenience sampling. Participants were recruited via professional networks and social media platforms (WhatsApp, Telegram, and X). The survey instrument, adapted from validated international tools, was reviewed by experts and pilot-tested with 15 pharmacists (Cronbach’s alpha = 0.81). Data were analyzed using SPSS v26. Chi-square tests and t-tests were used to examine relationships between readiness and demographic/professional factors. Significance was set at p < 0.05.

**Results:**

Pharmacists exhibited moderate knowledge (mean = 13.8/20) and positive attitudes but reported training gaps and regulatory barriers. Most respondents supported early integration of palliative care, yet only 21.7% achieved full knowledge scores. Readiness was significantly higher among female (χ^2^ = 6.12, p = 0.047) and urban pharmacists (χ^2^ = 8.76, p = 0.013), while emotional preparedness and communication comfort remained comparatively low.

**Conclusion:**

Community pharmacists in the Aseer region show strong willingness but limited preparedness for palliative care services. Addressing training deficiencies through structured modules and continuing education is essential. Developing national guidelines and targeted support programs will strengthen pharmacists’ roles and align their contributions with Vision 2030 healthcare goals.

## Introduction

Palliative care (PC) is a holistic approach designed to enhance the quality of life for patients with life-limiting diseases by addressing their physical, emotional, and spiritual needs. The World Health Organization (WHO) acknowledges palliative care as a fundamental human right and advocates for its incorporation into primary and community care services to guarantee equal access for all patients, particularly those with chronic and terminal conditions (Care, 2018). Pharmacists, particularly in community settings, are increasingly recognized as vital members of multidisciplinary palliative care teams due to their proficiency in medication management and patient counseling (Krzyżaniak et al., 2016).

Community pharmacists are ideally situated to assist patients near the end of life due to their accessibility and frequent encounters with patients and caregivers. Their responsibilities may encompass not only dispensing but also conducting drug reviews, deprescribing, managing intricate regimens, and providing education on opioid usage and symptom-relief medications (Thrimawithana et al., 2024). Nevertheless, despite this potential, pharmacists are frequently underrepresented in numerous palliative care approaches globally.

Several studies have demonstrated the efficacy of pharmacist-led interventions in community-based primary care settings. In the United Kingdom, Needham et al. (2002) found that 81% of clinical interventions conducted by trained community pharmacists were considered advantageous by a multidisciplinary expert panel, especially in enhancing symptom management and medication compliance. More recent studies, such as Tait et al. (2020) and Thrimawithana et al. (2024), confirm that pharmacists continue to face barriers worldwide, including insufficient training, unclear role definitions, and limited integration into interdisciplinary teams (Thrimawithana et al., 2024; Tait et al., 2020).

Furthermore, pharmacists can significantly contribute to easing the psychological distress of both patients and caregivers. In the research conducted by Jiwa et al. (2012), patients expressed increased confidence in their care when pharmacists performed home medication assessments. This support is crucial for addressing concerns associated with opioid usage, side effects, and polypharmacy (Jiwa et al., 2012).

Despite this evidence, integration persists in being inconsistent. Obstacles encompass restricted access to patient health records, inadequate standardized training in pharmaceutical care for pharmacists, insufficient policy support (Al-Jedai et al., 2016), and inefficient interdisciplinary teamwork (Thrimawithana et al., 2024; Akram et al., 2017). Moreover, cultural and religious sensitivities, especially around dialogues about mortality and the utilization of controlled substances, can limit pharmacist involvement, particularly in conservative societies.

The enhancement of palliative care services is a primary goal within the Saudi Arabia Vision 2030 healthcare transformation initiative (Alqahtani and Albabtain, 2025). Nonetheless, pharmacist involvement in palliative care is still in its infancy. Despite the growing acknowledgment of hospital-based clinical pharmacists’ services, community pharmacists, sometimes the initial point of contact, are seldom incorporated into official palliative care teams. Recent Saudi studies by Alshehri et al. (2023) indicate that pharmacists have limited exposure to palliative care and varying levels of knowledge and intention to provide such services (Alshehri et al., 2023). However, no study to date has comprehensively assessed community pharmacists’ readiness and barriers to delivering palliative care in Saudi Arabia, particularly in regional practice settings. This gap highlights the urgency of the present research. While the Ministry of Health has outlined national palliative care strategies, there are currently no specific Saudi guidelines defining the responsibilities of community pharmacists in this field. This absence of role-specific guidance underscores the importance of evaluating their current perceptions and readiness.

Therefore, this study aims to evaluate the perceptions, readiness, and barriers faced by community pharmacists in Saudi Arabia regarding their participation in palliative care provision. By situating these findings within global evidence and national healthcare priorities, we seek to inform educational development, workforce planning, and policy frameworks aligned with Vision 2030.

## Methods

### Study design and setting

This study employed a cross-sectional survey design to evaluate the perceptions, readiness, and perceived barriers among community pharmacists regarding their role in providing palliative care services. The research was conducted between January and April 2025 in the Aseer region of Saudi Arabia. Although earlier drafts suggested a broader scope, the study was limited to this region.

### Study population and sampling

The target population consisted of licensed community pharmacists operating in the Aseer region of Saudi Arabia. As per the most recent figures (11), more than 747 community pharmacists are presently registered in the region, with a substantial percentage employed in community settings. A convenience sampling method was employed to enlist participants using professional pharmaceutical networks and social media platforms (WhatsApp, Telegram, and X). Pharmacists not engaged in active practice or solely employed in hospital environments were excluded. Using Raosoft’s online sample size calculator, and assuming a 95% confidence interval, a 5% margin of error, and an estimated population of 747 community pharmacists, a minimum sample size of 254 was required to ensure statistical reliability. To maximize response rates, two reminder messages were sent at weeks two and four of the survey period.

### Survey instrument development

The survey instrument was adapted from validated tools used in previous international studies assessing pharmacists’ involvement in palliative care (Thrimawithana et al., 2024). Specifically, core items were drawn from previously validated questionnaires developed by Thrimawithana et al. (2024) and Needham et al. (2002), with contextual modifications to align with Saudi community pharmacy practice. Example knowledge items included statements such as “Palliative care can begin alongside curative treatment” and “Opioids should always be avoided due to their addiction potential” (reverse-coded). Attitude and readiness items reflected agreement with statements like “Pharmacists have a valuable role in palliative care” and “I am emotionally prepared to care for terminally ill patients.” Barrier items addressed training, collaboration, and legal limitations. It was composed of five sections.

1. Demographic characteristics (age, gender, qualifications, years of experience, region of practice);
2. Knowledge about palliative care and related clinical responsibilities;
3. Attitudes toward the provision of palliative services;
4. Self-assessed readiness to provide palliative care;
5. Perceived barriers to pharmacist involvement;

Items were structured utilizing 5-point Likert scales (e.g., strongly agree to strongly disagree) and multiple-choice questions. Knowledge items were scored as one point for each correct response, yielding a total knowledge score ranging from 0 to 20, which was also categorized as ‘adequate’ (≥70% correct) or ‘inadequate’ (<70%) for subgroup comparisons. Readiness items were rated on a 5-point Likert scale and averaged to generate a composite readiness index; higher mean scores reflected greater preparedness. For descriptive analysis, readiness was further classified into high, moderate, or low based on tertile distribution.

Content validity was confirmed via expert evaluation by a panel comprising clinical pharmacists, palliative care specialists, and academic researchers. A pilot test involving 15 community pharmacists was performed to verify clarity and reliability, yielding a Cronbach’s alpha of 0.81, which signifies strong internal consistency. Minor cultural tailoring was performed to ensure contextual appropriateness, including adjustments to terminology around opioid regulation and communication practices in line with Saudi healthcare norms. As English is the standard language of pharmacy education and practice in Saudi Arabia, no translation was required. A copy of the full questionnaire is provided as Supplementary Material (Appendix 1).

### Data collection procedures

The final survey was distributed electronically via Google Forms, with informed consent embedded at the beginning of the questionnaire. Participation was voluntary, and respondents were assured of the anonymity and confidentiality of their responses. The survey remained open for 6 weeks, with two reminder prompts circulated to enhance participation.

### Ethical considerations

Ethical approval was obtained from the Ethical Research Committee at King Khalid University (Approval Number: KKU-27-2025-14). All procedures adhered to the Declaration of Helsinki guidelines. No identifiable personal data was collected.

### Data analysis

Data were transferred to Microsoft Excel and analyzed utilizing SPSS version 26. Descriptive statistics, including frequencies and percentages, were employed to summarize the demographic and baseline characteristics. Inferential statistics, such as chi-square tests and independent t-tests, were performed to investigate the relationships between pharmacists’ preparedness and their demographic or professional attributes. A significance level of p < 0.05 was utilized for all analyses.

Data were transferred to Microsoft Excel and analyzed utilizing SPSS version 26. Descriptive statistics, including frequencies and percentages, were employed to summarize the demographic and baseline characteristics. Inferential statistics, such as chi-square tests and independent t-tests, were performed to investigate the relationships between pharmacists’ preparedness and their demographic or professional attributes. Chi-square was used to assess associations between categorical variables, while t-tests and ANOVA compared mean differences across continuous variables. Missing data were handled using pairwise deletion. Knowledge scores were computed as continuous variables (total score out of 20). For subgroup comparisons, knowledge was categorized as ‘adequate’ (≥70% correct responses) or ‘inadequate’ (<70%), consistent with thresholds reported in prior pharmacy education studies. A significance level of p < 0.05 was applied for all analyses.

Bivariate analyses (chi-square, t-test, and ANOVA) were selected to explore associations between readiness, knowledge, and demographic variables, as the study’s primary objective was to identify preliminary relationships rather than develop predictive models. Multivariate analysis was considered during the design stage but was not applied, as the number of variables and the moderate sample size (n = 267) limited the statistical power for stable regression modeling. This approach ensured clarity in interpretation and alignment with similar cross-sectional pharmacy readiness studies.

## Results

### Participant demographics

A total of 267 community pharmacists participated in the study, representing 35.7% of the target population in the Aseer region. The majority were male (72.7%), aged between 20 and 39 years (92.9%), and held a PharmD degree (77.9%). Nearly half (47.6%) had fewer than 5 years of professional experience, and most were practicing in urban settings (71.9%) (Table 1).

**TABLE 1:** Participants’ demographic characteristics.

Characteristic	Frequency (n)	Percentage (%)
Gender		
Male	194	72.7
Female	73	27.3
Age group		
20–29 years	135	50.6
30–39 years	113	42.3
40+ years	19	7.1
Education level		
Bachelor’s (BPharm)	51	19.1
PharmD	208	77.9
Postgraduate	8	3
Years of experience		
<5 years	127	47.6
5–10 years	90	33.7
>10 years	50	18.7
Practice location		
Urban	192	71.9
Rural	75	28.1

### Participants’ knowledge about palliative care

The overall mean knowledge score was 13.8 (SD = 3.6) out of 20, reflecting a moderate overall level of awareness. Most pharmacists recognized that palliative care can begin alongside curative treatment (74.2%), and more than half understood its role in addressing psychosocial needs (53.9%). However, substantial knowledge gaps remained, as only 21.7% correctly identified morphine as the opioid of choice for severe pain (Table 2). Knowledge scores were notably higher among pharmacists with more than 10 years of experience, while no significant differences were observed by gender or education level (as detailed in Table 6).

**TABLE 2:** Participants’ knowledge about palliative care.

Knowledge question	Correct answer	Correct responses (n, %)
Palliative care can begin alongside curative treatment	TRUE	198 (74.2%)
Palliative care is only for end-stage cancer patients	FALSE	144 (53.9%)
Pharmacists can manage drug-related problems in palliative care	TRUE	183 (68.5%)
Opioids should always be avoided due to their addiction potential	FALSE	162 (60.7%)
Correctly answered all knowledge items	All correct	58 (21.7%)

### Participants’ attitudes towards providing palliative care

Overall, respondents demonstrated positive attitudes toward the provision of palliative care, with most agreeing that community pharmacists should have an active role in pain and symptom management (60.7%) and patient counseling (55.4%) (Table 3). Nonetheless, fewer participants felt adequately emotionally prepared to provide end-of-life care (57.3%), highlighting a critical training gap. Attitudes were significantly associated with gender, with female pharmacists expressing more positive views than males, and with greater professional experience (as summarized in Table 6). Differences by age group and education level did not reach statistical significance.

**TABLE 3:** Participants’ attitudes toward providing palliative care services.

Attitude item	Strongly agree (n, %)	Agree (n, %)	Neutral (n, %)	Disagree (n, %)	Strongly disagree (n, %)
Pharmacists have a valuable role in palliative care	162 (60.7%)	78 (29.2%)	16 (6.0%)	7 (2.6%)	4 (1.5%)
Palliative care should be part of community pharmacy services	148 (55.4%)	90 (33.7%)	20 (7.5%)	6 (2.2%)	3 (1.1%)
I am willing to be part of a multidisciplinary palliative care team	153 (57.3%)	87 (32.6%)	17 (6.4%)	6 (2.2%)	4 (1.5%)
Pharmacists should receive structured training in palliative care	160 (59.9%)	83 (31.1%)	15 (5.6%)	7 (2.6%)	2 (0.7%)
Providing palliative care aligns with my professional responsibilities	150 (56.2%)	88 (33.0%)	20 (7.5%)	5 (1.9%)	4 (1.5%)
I am emotionally prepared to care for terminally ill patients	86 (32.2%)	83 (31.1%)	58 (21.7%)	30 (11.2%)	10 (3.7%)

Overall, pharmacists’ attitude scores demonstrated positive perceptions toward palliative care, with a composite mean of 4.1 ± 0.6 on the 5-point Likert scale. Tables 3–5 share a common Likert-scale structure but are presented individually to emphasize the differences between the attitudes, barriers, and readiness domains.

**TABLE 4:** Perceived barriers to providing palliative care.

Barrier	Strongly disgree (n, %)	Disagree (n, %)	Neutral (n, %)	Agree (n, %)	Strongly agree (n, %)
Lack of training in palliative care	12 (4.5%)	18 (6.7%)	18 (6.7%)	61 (22.8%)	158 (59.2%)
Lack of collaboration with physicians/healthcare teams	20 (7.5%)	23 (8.6%)	42 (15.7%)	74 (27.7%)	108 (40.4%)
Fear of mismanagement or legal consequences	28 (10.5%)	30 (11.2%)	48 (18.0%)	67 (25.1%)	94 (35.2%)
Time constraints or high workload	35 (13.1%)	40 (15.0%)	47 (17.6%)	68 (25.5%)	77 (28.8%)
Cultural discomfort discussing end-of-life issues	41 (15.4%)	38 (14.2%)	50 (18.7%)	63 (23.6%)	75 (28.1%)
No access to palliative care guidelines	21 (7.9%)	33 (12.4%)	40 (15.0%)	83 (31.1%)	90 (33.7%)
Inadequate compensation or recognition	55 (20.6%)	47 (17.6%)	59 (22.1%)	53 (19.9%)	53 (19.9%)

**TABLE 5:** Pharmacists’ readiness to provide palliative care.

Readiness statement	Strongly disgree (n, %)	Disagree (n, %)	Neutral (n, %)	Agree (n, %)	Strongly agree (n, %)
I feel confident in my ability to provide palliative care	12 (4.5%)	20 (7.5%)	55 (20.6%)	110 (41.2%)	70 (26.2%)
I am willing to be part of a multidisciplinary palliative care team	8 (3.0%)	18 (6.7%)	40 (15.0%)	120 (44.9%)	81 (30.3%)
I am emotionally prepared to deal with end-of-life patient needs	25 (9.4%)	35 (13.1%)	60 (22.5%)	95 (35.6%)	52 (19.5%)
I believe palliative care is part of my professional role as a pharmacist	6 (2.2%)	14 (5.2%)	44 (16.5%)	122 (45.7%)	81 (30.3%)
I am comfortable communicating with terminally ill patients and their families	30 (11.2%)	50 (18.7%)	70 (26.2%)	80 (30.0%)	37 (13.9%)
I have adequate knowledge to support patients in palliative care settings	15 (5.6%)	28 (10.5%)	60 (22.5%)	100 (37.5%)	64 (24.0%)

### Perceived barriers to palliative care involvement

The most commonly reported barriers were insufficient training (59.2%) and limited collaboration with other healthcare professionals (40.4%), followed by legal/clinical concerns (35.2%) and time constraints (28.8%) (Table 4). These findings underscore the structural and educational challenges limiting pharmacists’ contribution to palliative care. Barriers were more frequently reported by pharmacists with fewer than 5 years of experience and by those practicing in rural areas, whereas differences by education level were not statistically significant (Table 6).

**TABLE 6:** Relationship between community pharmacists’ demographic and professional characteristics and outcomes (Readiness, Knowledge, Attitudes, and Barriers).

Variable	Readiness high n (%)	Readiness moderate n (%)	Readiness low n (%)	Knowledge mean (SD)	Attitudes positive (%)	Barriers reported (%)	Chi-square/t/OR	p-value	95% CI (if applicable)
Gender							χ^2^ = 6.12/t = 1.75	0.047^a^/0.08	0.86–3.97
Male	85 (42.5%)	95 (47.5%)	20 (10.0%)	13.5 (3.6)	76.0	61.2			
Female	40 (55.0%)	25 (34.2%)	8 (11.0%)	13.9 (3.5)	85.0	66.5			
Age group							χ^2^ = 9.45	0.051	
20–29 years	70 (51.9%)	50 (37.0%)	15 (11.1%)	13.3 (3.7)	75.0	68.0			
30–39 years	45 (40.5%)	55 (49.5%)	11 (10.0%)	13.6 (3.5)	79.0	64.0			
40+ years	10 (52.6%)	7 (36.8%)	2 (10.5%)	14.2 (3.3)	84.0	60.0			
Education level							χ^2^ = 7.89	0.096	
BPharm	18 (30.5%)	30 (50.8%)	11 (18.7%)	13.4 (3.7)	78.0	64.0			
PharmD	97 (46.4%)	90 (43.1%)	22 (10.5%)	13.7 (3.6)	80.0	62.0			
Postgraduate	5 (62.5%)	3 (37.5%)	0 (0.0%)	14.1 (3.1)	90.0	60.0			
Years of experience							χ^2^ = 12.34/t = 3.12	0.015^a^/0.002	1.2–3.0
<5 years	45 (35.4%)	65 (51.2%)	17 (13.4%)	12.9 (3.7)	74.0	72.0			
5–10 years	50 (52.6%)	38 (40.0%)	7 (7.4%)	13.6 (3.4)	80.0	65.0			
>10 years	30 (60.0%)	17 (34.0%)	3 (6.0%)	14.7 (3.2)	86.0	55.0			
Practice location							χ^2^ = 8.76	0.013^a^	
Urban	100 (52.1%)	75 (39.1%)	17 (8.8%)	13.9 (3.5)	83.0	58.0			
Rural	25 (32.0%)	45 (57.7%)	8 (10.3%)	13.2 (3.8)	75.0	70.0			

^a^ P-value <0.05 indicates statistically significant.

Overall, pharmacists reported moderate-to-high agreement with identified barriers, with a mean composite score of approximately 3.9 ± 0.7 on the 5-point scale, dominated by training-related gaps and limited collaboration.

### Pharmacists’ readiness to provide palliative care

Table 5 summarizes pharmacists’ readiness to engage in palliative care. The majority expressed confidence in their competence and willingness to participate in a multidisciplinary team, although emotional preparedness and comfort with end-of-life communication were comparatively lower. Agreement was highest for viewing palliative care as part of their professional role (76.0%) and lowest for emotional readiness (55.1%). Readiness showed significant associations with gender, years of professional experience, and practice location, where female, more experienced, and urban pharmacists demonstrated higher readiness (as detailed in Table 6).

The overall mean readiness score was 3.8 ± 0.6, indicating moderate preparedness among participants.

### Factors associated with readiness to provide palliative care

Table 6 illustrates the correlation between pharmacists’ demographic and professional attributes and their readiness to provide palliative care. Significant correlations were observed for gender (χ^2^ = 6.12, p = 0.047), years of professional experience (χ^2^ = 12.34, p = 0.015; t = 3.12, p = 0.002, 95% CI: 1.2–3.0), and practice location (χ^2^ = 8.76, p = 0.013). Female pharmacists demonstrated higher readiness, with about 55% classified as having strong readiness compared with about 43% of males. Those with more than 10 years of experience were more likely to be highly prepared (about 60% *versus* about 35% among pharmacists with less than 5 years’ experience). Urban pharmacists also reported higher readiness than rural colleagues (around 52% *versus* about 32%). No statistically significant associations were found for age group (χ^2^ = 9.45, p = 0.051) or education level (χ^2^ = 7.89, p = 0.096), although descriptive trends suggested slightly higher readiness among younger pharmacists and those with postgraduate degrees. An asterisk (*) denotes statistical significance at p < 0.05.

## Discussion

This study provides the first regional assessment of community pharmacists’ knowledge, attitudes, emotional preparedness, and barriers to delivering palliative care in the Aseer region of Saudi Arabia. Key findings include moderate knowledge levels, positive overall attitudes, significant gaps in emotional readiness, and demographic associations showing greater readiness among female, experienced, and urban pharmacists. In accordance with Alshehri et al. (2023), who employed the Theory of Planned Behaviour (TPB), our results indicate that pharmacists had robust intentions and positive attitudes about palliative care (Alshehri et al., 2023). Nonetheless, information gaps remain, as many misconstrue the extent of palliative care beyond end-of-life services, echoing the findings of Alshehri et al. about analogous misconceptions (Akram et al., 2017). Miller (2017) recognized insufficient training as a universal challenge to pharmacists’ participation in palliative care (Alshehri et al., 2023). These findings underscore the necessity for enhanced education to elevate clinical competence.

The observed gender differences in readiness may reflect underlying social and professional dynamics in Saudi community pharmacy practice. Female pharmacists often engage more in patient counseling, communication, and chronic care management, roles that foster empathy and interpersonal connection traits associated with higher palliative care readiness (Alshahrani, 2025; Alsolami et al., 2023). Additionally, female practitioners may have greater exposure to counseling responsibilities within community pharmacies, further strengthening these skills. Location-based variations, with higher readiness among urban pharmacists, can be explained by their increased access to training, hospital collaboration, and exposure to interdisciplinary teams compared with rural counterparts. These differences highlight the influence of both workplace structure and professional environment on readiness outcomes (Alsolami et al., 2023).

Primary barriers encompass inadequate training and restricted engagement with healthcare practitioners, aligning with Alshehri et al.'s assertion that the majority of pharmacists have minimal prior expertise in palliative care (Alshehri et al., 2023). Regulatory limitations, especially strict opioid dispensing regulations, restrict participation, supporting Aleid et al. (2024) examination of opioid prescribing concerns (Aleid et al., 2024). Miller (2017) and Cuchet et al. (2024) identified regulatory and structural barriers, noting that French pharmacists have analogous collaborative difficulties in palliative home care (Miller, 2017; Cuchet et al., 2024). On the contrary, Australian pharmacists get advantages from integrated responsibilities, as demonstrated by Hussainy et al. (2011) and Thrimawithana et al. (2024). The World Health Organization (2018) endorses policy frameworks for the incorporation of pharmacists into palliative care (Care, 2018), suggesting that national guidelines in Saudi Arabia, along with continuing education (CE), could mitigate these obstacles and augment pharmacists’ roles.

The present study demonstrated significant willingness (75.2%) and confidence (67.4%) among Saudi community pharmacists to participate in palliative care, consistent with worldwide findings from Australia and the United States (O'Connor et al., 2013; Herndon et al., 2016). However, emotional preparedness (55.1%) and communication comfort (43.9%) were considerably lower. This may reflect cultural sensitivities surrounding end-of-life discussions, limited exposure to palliative cases in practice, and a lack of structured psychosocial training in pharmacy curricula. These factors collectively may inhibit pharmacists’ confidence when communicating with patients and families during palliative care. These results align with previous findings by Flannery et al. (2022), which highlighted deficiencies in psychosocial competencies (Flannery et al., 2022). The 61.5% indicating sufficient understanding reflects global patterns of intermediate preparedness, with persistent need across studies for enhanced training and clearer role delineation (Thomas et al., 2024).

Within the Saudi cultural and ethical context, communication about death and terminal illness can be highly sensitive, influenced by religious and societal values emphasizing hope, family protection, and collective decision-making (Mani, 2024; Glyn-Blanco et al., 2023). Pharmacists may therefore feel constrained in addressing end-of-life topics directly, which can affect emotional readiness and communication confidence. Incorporating cultural competence and ethical communication frameworks into continuing education programs could help pharmacists navigate these conversations respectfully while maintaining professional compassion and patient autonomy (Mani, 2024; Glyn-Blanco et al., 2023).

Female pharmacists may experience greater readiness due to gender-related social factors that encourage communication and patient counseling. Similarly, pharmacists with more years of practice and those working in urban locations likely have greater exposure to multidisciplinary teams and clinical resources, which enhances readiness. These patterns suggest that workforce planning should prioritize mentoring and targeted training for rural and early-career pharmacists.

Unlike previous Saudi studies focused largely on hospital pharmacists, this is the first to evaluate community pharmacists’ readiness and to quantify emotional preparedness as a distinct domain.

Global comparisons indicate shared difficulties. O’Connor et al. (2013) and Tait et al. (2013) identified training and collaboration barriers among Australian pharmacists, but Saudi pharmacists encounter stricter regulatory challenges (O'Connor et al., 2013; Tait et al., 2013). Miller (2017), Thrimawithana et al. (2024), and Cuchet et al. (2024) recognized worldwide difficulties of time limits, guideline deficiencies, and interprofessional coordination challenges (Thrimawithana et al., 2024; Miller, 2017; Cuchet et al., 2024). Alshehri et al. (2023) emphasized the impact of favorable subjective norms on intentions, a feature that has received less examination in this context but is essential for collaboration. Our research distinctly highlights the emotional challenges of palliative care, as numerous pharmacists indicate insufficient emotional readiness, a deficiency not acknowledged by Alshehri et al. (2023). CE programs that integrate psychosocial skills, as proposed by Thrimawithana et al. (2024) and Cuchet et al. (2024), may effectively address this issue (Thrimawithana et al., 2024; Cuchet et al., 2024).

Continuing education is crucial for addressing knowledge deficiencies, with pharmacists advocating for mandatory training modules. Alshehri et al. determined that the completion of a pharmacy residency program was associated with enhanced intentions to deliver palliative care (Alshehri et al., 2023). The Saudi Commission for Health Specialties (SCFHS) requires continuing education for license renewal; nevertheless, there is an absence of modules specifically focused on palliative care. International evidence reinforces these implementation strategies. Studies in Australia, the UK, and France demonstrated that focused continuing education enhanced pharmacists’ confidence and clinical contributions to palliative care (Needham et al., 2002; Cuchet et al., 2024; Hussainy et al., 2011). CE has also shown benefits in cancer pain management (Edwards et al., 2019) and symptom control [3].

Strengthening these initiatives aligns directly with Saudi Vision 2030, which prioritizes community-based healthcare delivery, workforce empowerment, and enhanced patient-centered services (Alqahtani and Albabtain, 2025; Almobarak, 2024). Integrating palliative care into community pharmacy practice supports Vision 2030s objectives to decentralize services and improve accessibility across all regions. Furthermore, aligning Saudi pharmacy practice with the WHO (2018) recommendation for integrating palliative care into primary healthcare and with international models such as those established in Australia and the UK could serve as a strategic framework for national policy advancement (Alhatim et al., 2024; Care, 2018).

Saudi Arabia’s healthcare transformation under Vision 2030 creates opportunities for pharmacists to reduce hospital burdens and enhance community-based palliative care (Alshahrani et al., 2021). With a mix of urban and rural practitioners, pharmacists are well-positioned to improve access in underserved regions. These findings directly intersect with Vision 2030 goals, which emphasize shifting care toward community settings and reducing hospital burden. The persistent training gaps and regulatory restrictions we observed represent obstacles to those national goals, whereas pharmacists’ willingness to provide palliative care highlights a workforce opportunity to advance Vision 2030 objectives. Collaboration frameworks are essential for integrating pharmacists into multidisciplinary teams, with CE fostering interprofessional skills. The WHO (2018) emphasizes integrating palliative care into primary healthcare, a model that could guide Saudi policy development (Care, 2018).

Despite its strengths, this study is subject to several limitations. The use of a convenience sampling approach may have introduced selection bias, as participation was likely influenced by pharmacists with prior interest in palliative care or professional development. The study’s focus on the Aseer region limits the generalizability of findings to other regions of Saudi Arabia with differing practice environments and demographic characteristics. Furthermore, the cross-sectional and quantitative design restricts the ability to explore underlying motivations, cultural nuances, or emotional challenges in depth. As with any self-reported survey, social desirability bias may have inflated perceived readiness. Knowledge items, though pilot-tested, may still be misinterpreted. In addition, the urban-heavy sample limits generalizability to rural pharmacists, and the cross-sectional design precludes causal inference. The significant prevalence of PharmD holders may not accurately represent the comprehensive educational background of Saudi pharmacists, perhaps exaggerating their readiness in comparison to the group studied by Alshehri et al. (2023). The dissemination of surveys through pharmacy networks involves the risk of selection bias, as it is probable that only motivated pharmacists participated, a concern highlighted by Alshehri et al. (2023). The absence of qualitative data obstructs understanding of emotional or regulatory difficulties, indicating the need for mixed-methods approaches in future studies. Unassessed CE programs restrict insights into effectiveness; however, Needham et al. (2002) and Cuchet et al. (2024) underscore the usefulness of CE (Needham et al., 2002; Cuchet et al., 2024). Omitting hospital pharmacists neglects context-specific preparedness, as indicated by Thrimawithana et al. (2024), who propose varied functions (Thrimawithana et al., 2024). Future research should include qualitative interviews to explore cultural and emotional barriers, pilot interventional studies testing CE modules, and comparative studies of rural *versus* urban or hospital *versus* community pharmacists. Longitudinal designs are also needed to evaluate the sustained impact of training under Vision 2030. Although bivariate analyses were appropriate for the study’s scope, future research should employ regression or multivariate models to better identify independent predictors of pharmacists’ readiness and knowledge.

## Conclusion

This study provides the first regional (Aseer) assessment of community pharmacists’ readiness to deliver palliative care services in Saudi Arabia. By evaluating knowledge, attitudes, emotional preparedness, and perceived barriers, the study offers new insights into the underexplored role of community pharmacists in palliative care. Importantly, it is also the first Saudi study to quantify emotional preparedness as a key dimension of readiness.

Findings demonstrate that pharmacists possess a strong willingness to contribute to palliative care, yet face significant barriers, including limited training, regulatory constraints, and low emotional preparedness. Female, more experienced, and urban pharmacists exhibited greater readiness, suggesting disparities that require attention in workforce planning. While these results highlight opportunities for expanding the role of community pharmacists in line with Saudi Arabia’s Vision 2030, the regional nature of this study necessitates caution in generalization, and replication in other regions is essential before drawing national conclusions.

Future research should pursue qualitative studies to explore cultural and emotional barriers, pilot continuing-education interventions, and longitudinal evaluations of pharmacist-led palliative care services. Comparative studies across rural and urban areas, as well as hospital *versus* community pharmacy settings, are also recommended.

In terms of practical implications, the findings underscore the need to integrate structured palliative care and psychosocial modules into pharmacy curricula and continuing education, to develop national guidelines clarifying the role of community pharmacists in palliative care and strengthening collaboration frameworks, and to foster interprofessional practice models supported by online education platforms to reach rural pharmacists. Specifically, pharmacy schools should embed mandatory palliative care and communication modules within undergraduate programs to build foundational competence. National health authorities and the Saudi Commission for Health Specialties should implement accredited continuing-education programs focusing on symptom management and ethical communication. Additionally, policymakers should revise existing pharmacy regulations to formally recognize and expand pharmacists’ roles in multidisciplinary palliative care delivery, in alignment with Vision 2030 healthcare transformation objectives. Future nationwide studies utilizing advanced statistical modeling could further validate these findings and support evidence-based workforce and policy reforms aligned with Saudi Vision 2030.

## Data Availability

The raw data supporting the conclusions of this article will be made available by the authors, without undue reservation.
