# Supplementary material for: Perception and readiness of community pharmacists in delivering palliative care services in Saudi Arabia: a new role in the game
Source: Front Pharmacol. 2025 Nov 25;16:1646531. doi: 10.3389/fphar.2025.1646531 (PMC12685817; doi:10.3389/fphar.2025.1646531)
Supplement: Supplementary file 1 [file DataSheet1.pdf]

Perception and Readiness of Community Pharmacists in Delivering Palliative Care Services in Saudi Arabia: A New Role in the Game

We are a group of researchers in King Khalid University conducting a research project entitled " Perception and Readiness of Community Pharmacists in Delivering Palliative Care Services in Saudi Arabia: A New Role in the Game". The purpose of this project is to explore the Perception and Readiness of Community Pharmacists in Delivering Palliative Care Services in Saudi Arabia. Your participation in this research project is voluntary. You may choose not to participate. If you decide to participate in this research survey, you may withdraw at any time. The procedure involves filling a survey that will take approximately 3-5 minutes. Your responses will be confidential, and we do not collect identifying information such as your name, email address or IP address.

Survey:

Section A: Demographic and Professional Information

1. **Age group**: ☐ 20–29 years ☐ 30–39 years ☐ 40+ years
2. **Gender**: ☐ Male ☐ Female
3. **Years of professional experience**
   ☐ Less than 5 years ☐ 5–10 years ☐ More than 10 years
4. **Highest Qualification**:
   ☐ BPharm ☐ PharmD ☐ Master's or PhD
5. **Practice location**: ☐ Urban ☐ Rural

Section B: Knowledge of Palliative Care *(T:True/F:False)*

Statement	T	F
Palliative care can begin alongside curative treatment. (T)	☐	☐
Palliative care is only for end-stage cancer patients. (F)	☐	☐
Pharmacists can manage drug-related problems in palliative care. (T)	☐	☐
Opioids should always be avoided due to their addiction potential. (F)	☐	☐

Section C: Attitudes Toward Palliative Care *(Likert Scale: 1 = Strongly Disagree to 5 = Strongly Agree)*

Statement	1	2	3	4	5
I believe pharmacists have a valuable role in palliative care.	☐	☐	☐	☐	☐
Palliative care should be part of community pharmacy services.	☐	☐	☐	☐	☐
I am willing to be part of a multidisciplinary palliative care team.	☐	☐	☐	☐	☐
Pharmacists should receive structured training in palliative care.	☐	☐	☐	☐	☐
Providing palliative care aligns with my professional responsibilities.	☐	☐	☐	☐	☐
I am emotionally prepared to care for terminally ill patients.	☐	☐	☐	☐	☐

Section D: Perceived barriers *(Likert Scale: 1 = Strongly Disagree to 5 = Strongly Agree)*

Statement	1	2	3	4	5
Lack of training in palliative care limits my involvement.	☐	☐	☐	☐	☐
Lack of collaboration with physicians/healthcare teams is a barrier.	☐	☐	☐	☐	☐
I fear mismanagement or legal consequences in palliative care.	☐	☐	☐	☐	☐
Time constraints or workload hinder my ability to provide palliative care.	☐	☐	☐	☐	☐
Cultural discomfort discussing end-of-life issues is a barrier.	☐	☐	☐	☐	☐
Lack of access to palliative care guidelines limits my participation.	☐	☐	☐	☐	☐
Inadequate compensation or recognition discourages involvement.	☐	☐	☐	☐	☐

Section E: Barriers to Participation in Palliative Care *(Likert Scale: 1 = Strongly Disagree to 5 = Strongly Agree)*

Barrier	1	2	3	4	5
I feel confident in my ability to provide palliative care.	☐	☐	☐	☐	☐
I am willing to be part of a multidisciplinary palliative care team.	☐	☐	☐	☐	☐
I am emotionally prepared to deal with end-of-life patient needs.	☐	☐	☐	☐	☐
I believe palliative care is part of my professional role as a pharmacist.	☐	☐	☐	☐	☐
I am comfortable communicating with terminally ill patients and their families.	☐	☐	☐	☐	☐
I have adequate knowledge to support patients in palliative care settings.	☐	☐	☐	☐	☐

Section F: Opportunities and Recommendations

1. **Would you participate in a CPD-accredited palliative care workshop?**
   ☐ Yes ☐ No ☐ Maybe
2. **In your opinion, what is the most critical role of a community pharmacist in palliative care?**
   (Short answer) ____
